# Congenital Cytomegalovirus Infection: Update on Diagnosis and Treatment

**DOI:** 10.3390/microorganisms8101516

**Published:** 2020-10-01

**Authors:** Giulia Chiopris, Piero Veronese, Francesca Cusenza, Michela Procaccianti, Serafina Perrone, Valeria Daccò, Carla Colombo, Susanna Esposito

**Affiliations:** 1 Paediatric Clinic Pietro Barilla Children’s Hospital, Department of Medicine and Surgery, University of Parma, 43121 Parma, Italy; giulia.chiopris@gmail.com (G.C.); piero.veronese@studenti.unipr.it (P.V.); francescacusenza7@gmail.com (F.C.); michela.procaccianti@outlook.it (M.P.); 2 Neonatology Clinic, Pietro Barilla Children’s Hospital, Department of Medicine and Surgery, University of Parma, 43121 Parma, Italy; serafina.perrone@unipr.it; 3 Cystic Fibrosis Unit, Department of Pathophysiology and Transplantation, Università degli Studi di Milano, 20122 Milan, Italy; valeria.dacco@policlinico.mi.it (V.D.); carla.colombo@unimi.it (C.C.)

**Keywords:** antiviral therapy, cytomegalovirus, congenital CMV, neonatal infection, vertical transmission

## Abstract

Congenital cytomegalovirus (cCMV) infection is the most common congenital viral infection and is the leading non-genetic cause of sensorineural hearing loss (SNLH) and an important cause of neurodevelopmental disabilities. The risk of intrauterine transmission is highest when primary infection occurs during pregnancy, with a higher rate of vertical transmission in mothers with older gestational age at infection, while the risk of adverse fetal effects significantly increases if fetal infection occurs during the first half of pregnancy. Despite its prevalence and morbidity among the neonatal population, there is not yet a standardized diagnostic test and therapeutic approach for cCMV infection. This narrative review aims to explore the latest developments in the diagnosis and treatment of cCMV infection. Literature analysis shows that preventive interventions other than behavioral measures during pregnancy are still lacking, although many clinical trials are currently ongoing to formulate a vaccination for women before pregnancy. Currently, we recommend using a PCR assay in blood, urine, and saliva in neonates with suspected cCMV infection. At present, there is no evidence of the benefit of antiviral therapy in asymptomatic infants. In the case of symptomatic cCMV, we actually recommend treatment with oral valganciclovir for a duration of 12 months. The effectiveness and tolerability of this therapy option have proven effective for hearing and neurodevelopmental long-term outcomes. Valganciclovir is reserved for congenitally-infected neonates with the symptomatic disease at birth, such as microcephaly, intracranial calcifications, abnormal cerebrospinal fluid index, chorioretinitis, or sensorineural hearing loss. Treatment with antiviral drugs is not routinely recommended for neonates with the mildly symptomatic disease at birth, for neonates under 32 weeks of gestational age, or for infants more than 30 days old because of insufficient evidence from studies. However, since these populations represent the vast majority of neonates and infants with cCMV infection and they are at risk of developing late-onset sequelae, a biomarker able to predict long-term sequelae should also be found to justify starting treatment and reducing the burden of CMV-related complications.

## 1. Background

Cytomegalovirus (CMV) is a double-stranded DNA virus and is a member of the *Herpesviridae* family. Infection with CMV is ubiquitous, infecting approximately half of the population in high-income countries by adulthood and nearly everyone by an early childhood in low- and middle-income countries [1,2]. CMV infection passes undetected in healthy children and adults. However, several high-risk groups, including immunocompromised organ transplant recipients, hematopoietic stem cell transplant recipients, and individuals infected with human immunodeficiency virus (HIV), are at risk of developing life-threatening and sight-threatening CMV disease [3]. CMV is also a major cause of morbidity and occasional mortality in neonates. In recent years, it has become evident that congenital CMV (cCMV) infection is the most common congenital viral infection, with an estimated birth prevalence of 0.2–6% in industrialized countries [4], while limited studies in developing countries (Asia, Africa, Latin America) have shown a prevalence ranging from 0.6% to 6.1% [5]. cCMV contributes to a high burden of disease and is the leading non-genetic cause of sensorineural hearing loss (SNLH) and an important cause of neurodevelopmental disabilities in children [6,7,8].

Intrauterine CMV transmission may occur in mothers without pre-existing immunity who first acquired CMV infection in pregnancy (primary infection) or in women with pre-existing antibodies to CMV either by reactivation of a previous maternal infection or by the acquisition of a different viral strain (non-primary infection) [9]. The risk of intrauterine transmission is highest when primary infection occurs during pregnancy, with a higher rate of vertical transmission in mothers with older gestational age at infection, while the risk of adverse fetal effects significantly increases if fetal infection occurs during the first half of pregnancy [10,11,12,13].

An important determinant of cCMV is the prevalence of maternal CMV infection in the population. Among low seroprevalence populations, one-half to three-quarters of all congenital infections among newborns are due to non-primary infection during pregnancy, whereas in populations with high maternal seroimmunity, almost all congenital infections occur as a result of non-primary infection. Therefore, countries with high seroprevalence have high rates of congenital infection, even though the risk of infecting the fetus is higher in cases of primary infection [14]. Moreover, some progress has been made in treating symptomatic newborns with cCMV. This narrative review aims to explore the latest developments in the diagnosis and treatment of congenital CMV infection.

## 2. Clinical Manifestation

Congenital infection may be classified as symptomatic or asymptomatic, even though there is no clear definition of these two classifications in the literature. In general, moderately to severely symptomatic cCMV is defined as infants who are infected and who have multiple manifestations or central nervous system (CNS) involvement; mildly symptomatic cCMV is defined as infants who have one or two isolated manifestations that are mild and transient; asymptomatic cCMV with isolated sensorineural hearing loss (SNHL) is defined as infants who have no apparent clinical symptoms other than hearing loss; asymptomatic cCMV is defined as infants who have no apparent abnormalities at birth and have normal hearing [15]. Among congenitally-infected infants, approximately 10–15% have signs and symptoms of the disease at birth, and approximately half experience long-term sequelae [16,17]. Among asymptomatic infants with cCMV, an estimated 10–15% will develop long-term sequelae. The most common long-term sequela is hearing loss [8,18].

Possible signs and symptoms associated with cCMV are explained in Table 1.

CCMV infection is the leading non-genetic cause of SNHL in children across studies. Among infants who develop CMV-related SNHL, hearing loss may be present at birth or may have delayed onset, occurring throughout the first several years of life. Approximately 50% of children with SNHL experience further deterioration or progression of their loss during childhood, and the degree of hearing loss may fluctuate in up to half of the infants [19]. Therefore, it is important that all infants with cCMV infection, irrespective of their clinical presentation at birth, receive serial audiological monitoring throughout the first years of life to promptly detect possible SNHL to proceed with non-pharmacological interventions that can reduce the functional impairment resulting from hearing loss, significantly improving the receptive and expressive language and the social-emotional development of the affected child [15,20,21].

Furthermore, cCMV is the leading viral cause of neurodevelopmental delay, with a large proportion of symptomatic infants suffering from some degree of psychomotor and cognitive disabilities and with visual impairment presenting in up to half of the symptomatic infants [7,22,23,24,25,26]. As many affected children require significant ongoing care and special therapeutic and educational services, the economic burden associated with congenital CMV infection is substantial.

## 3. Maternal Screening and Diagnosis

Considering that the majority of cCMV-infected children are born to CMV IgG-seropositive women (non-primary maternal infection), as demonstrated by population studies, international consensus discourages prenatal screening of pregnant women since it can lead to anxiety, additional tests, and even unnecessary termination of pregnancies [27,28,29,30,31]. However, a variable proportion of pregnant women is tested for CMV IgG and IgM antibodies in Europe [32]. In Italy, approximately 40% of pregnant women are tested for CMV based on their gynecologist’s or general practitioner’s recommendation [33]. Education of all pregnant women irrespective of their serostatus and screening of all newborns at birth represents more effective tools to prevent and identify cCMV-infected children [34]. Although thousands of children born each year suffer from a permanent disability due to cCMV, most pregnant women have not heard of CMV infection and its associated sequelae. CMV awareness rates (13–60%) are higher in Europe than in the United States, although CMV knowledge gaps exist [35,36,37].

Primary maternal infections can be identified by serologic testing using IgG and IgM serology: IgG avidity testing should be used only if CMV-specific IgM antibodies are positive. It is possible to discriminate between primary and non-primary CMV infections considering IgM-positive results in combination with IgG avidity results [38]. Low avidity is indicative of recent infection, while high IgG avidity early in pregnancy suggests primary infection occurring before conception [38,39]. However, sometimes testing early in pregnancy may reveal a CMV IgG avidity result in the grey zone, making consultation with parents challenging. A recent study showed that the risk of CMV transmission in pregnant women with IgG avidity in the grey zone during the first trimester of pregnancy was higher than that in infants born after non-primary infection, but more studies are needed to confirm this result [40].

A challenge for the clinician may be the discovery of low levels of CMV-IgG antibodies in maternal serum samples in the absence of IgM because this may be due to a true positive or a false-positive result. These women should be further investigated with additional tests to confirm the presence of CMV-specific antibodies. False-positive results are of great concern, considering that seronegative women can actually lower the risk of acquiring a primary infection when properly counseled about hygiene measures [41]. CMV-IgG avidity testing should not be performed on serum samples with low IgG levels, as these can give inappropriate IgG avidity results. An algorithm for dealing with low positive IgG samples may be needed, and in the absence of a gold standard method, an equivocal IgG serologic assay result in a pregnant woman should be considered negative to ensure that these women are assigned to the highest CMV risk group for pregnancy outcome [42].

## 4. Diagnosis of Fetal CMV Infection

Ultrasound imaging is useful to predict the prognosis of fetal infection, even though it has poor sensitivity in prenatal diagnosis [43]. Amniocentesis to perform PCR for CMV DNA is the best available prenatal diagnostic tool [44] since the infected fetus excretes urine containing the virus into the amniotic fluid. Two studies demonstrated that in the case of primary CMV infection, persisting levels of maternal DNAemia at the time of amniocentesis correlated with a high risk of CMV transmission to the fetus [45,46]. This method has high sensitivity and specificity when performed after 20–21 weeks of gestation and 8 weeks after estimated maternal seroconversion [1,47]. In this case, the prognostic evaluation of fetal infection relies on imaging using a combination of ultrasound and cerebral magnetic resonance imaging (MRI). Several studies identified a residual risk of hearing loss at birth when imaging examination was considered negative [48,49,50,51,52,53].

## 5. Neonatal Screening and Diagnosis

Because SNHL occurs in approximately 10–15% of infants with asymptomatic congenital CMV infection and most children will experience late-onset audiologic, neurologic, and developmental sequelae, neither physical examination at birth nor newborn hearing screening represent a trustworthy tool to identify children at risk; therefore, a reliable, rapid, and possibly not expensive method to screen newborns for congenital CMV infection is urgently needed. Early identification of these patients will allow them to be properly monitored and to intervene during the acquisition of speech and language skills [16]. The main challenge in diagnosing CMV at the perinatal stage is to distinguish between congenital and postnatal infections.

Any newborn with signs or symptoms indicative of intrauterine CMV infection should be tested [54]. Infected infants shed large amounts of virus in saliva and urine; therefore, these specimens are both useful for the identification of cCMV in infants. A variety of methods have been evaluated for use in the diagnosis of congenital CMV infection based on saliva, urine, and dried-blood-spot (DBS) specimens obtained from newborns. In particular, the usefulness of DBS specimens has been evaluated since this sample is routinely obtained in all infants, although for diagnostic purposes, this sample seems to have low sensitivity [55,56,57,58,59,60,61,62,63,64].

Virus isolation by culture from urine or saliva has long been the standard method for diagnosing cCMV infection. Nonetheless, this technique is expensive, requires tissue cultures, is not easily amenable to automation, and, therefore, cannot be adapted for large-scale newborn screening. Since PCR exhibits high sensitivity in both saliva and urine samples [61,65], postnatal diagnosis of cCMV is preferably performed via real-time polymerase chain reaction (PCR). Additional advantages of PCR assays are that assays are amenable to automation, are low-cost, are not affected by storage and transport conditions, and do not require maintenance of tissue culture facilities.

Saliva specimens are good for screening for cCMV; however, they are not routinely collected from neonates; therefore, a change in infrastructure is needed before this method could be used on a large scale [42]. Clinicians should be careful about testing saliva in the delivery room because this may increase the risk of false positives from cervicovaginal secretions; saliva should also be obtained at least 1 h after breastfeeding.

The other useful specimen in diagnostic congenital infection is urine because infected newborns shed a large amount of CMV in urine, but its collection using a bag may be complicated by several factors, such as inadequate diuresis, loss of samples, or contamination. The use of other strategies to collect urine, such as cotton balls or filter cards in diapers, has not been evaluated in large, population-based screening programs and has not been compared with a gold-standard diagnostic method [64,66].

In almost all countries, the screening program at birth has become a routine procedure to screen metabolic and genetic diseases in newborns, and DBSs on filter paper have been considered an interesting and practical specimen for the detection of congenital CMV infection. Barbi et al. first showed that extracting CMV DNA from DBS and successive amplification with real-time PCR (RT-PCR) could allow for the diagnosis of cCMV infection: the authors collected Guthrie cards from infants who were already tested positive for CMV infection with serological testing, finding a sensitivity up to 100% [67]; four years later, they confirmed their method’s high sensitivity and specificity, suggesting that it could represent a useful alternative to viral culture [55]. Since then, the use of DBS as a diagnostic tool for cCMV has been largely studied, but in the literature, we have found few supportive studies. The CMV and Hearing Multicenter Screening (CHIMES) study conducted by the National Institute on Deafness and Other Communication Disorders (NIDCD) in 2010 was the only study that compared CMV DNA testing by real-time PCR on Guthrie Card with a viral culture method using saliva, using two different protocols to extract DNA [60]. Overall, the sensitivity of DBS RT-PCR ranged from 28.3% to 34.4%, depending on the protocol. The specificity of both PCR protocols was 99.9%. These data demonstrated that RT-PCR analysis on DBS had low sensitivity for identifying newborns with cCMV infection since up to 80% of infants with congenital CMV infections could be missed; therefore, it could not be recommended as a mass screening tool for the diagnosis of cCMV. The high positive likelihood ratio (LR) for both PCR assays suggested strong evidence that a positive DBS PCR result could identify infants with cCMV infection. On the other hand, the negative LR was not low enough to exclude cCMV infection in newborns with a negative DBS PCR test. This study concluded that with the current methods, CMV testing with DBS RT-PCR is inappropriate for CMV screening, and its main application remains the retrospective diagnosis of cCMV infection in children with delayed-onset sequelae. In these populations, however, a negative result does not exclude cCMV infection.

In the literature, other studies can be found that describe higher sensitivity of detecting CMV DNA by PCR using DBSs, from 45% up to 82%, on average [59,68]; the highest detection rate was described by Barbi et al., whose study reached a 100% sensitivity. Nevertheless, all the above are retrospective studies or prospective studies conducted on selected populations with known CMV infection [67]. One of the reasons for the disappointing and variable sensibility of DBSs in the literature may be due to different technical assays (DNA extraction method, PCR protocol) [69] but is mainly due to the fact that newborns may have acquired the infection months previously, and thus, up to 10–20% of children may have non-detectable viremia at birth [54,61]. Another issue concerning the use of DBS for the diagnosis of cCMV infection is the fact that DBS samples are sometimes available for a short period. Wang et al. reported that the storage times of Guthrie cards vary from 14 days to 18 years among 10 countries [70]. For certain regions, some regulatory changes in political decision-making should be considered to allow for longer storage of these samples. Despite all of this, some authors provided evidence of the high sensitivity of DBS for the retrospective diagnosis of cCMV in children developing neurological sequelae in the first few years of life. Moreover, it is clear that some serious health problems (such as SNHL, cortical development, pachygyria, cholestasis) would have been lessened as a result of DBS retrospective diagnosis [71,72]. Finally, some authors reported the usefulness of DBS to identify children at risk of developing long-term sequelae [73,74].

A large prospective study published in 2017 found that PCR of DBS had low sensitivity and specificity for identifying infants with CMV-associated hearing loss, both at birth and at four years of age [63]. In conclusion, the role of DBS PCR for testing CMV is more established in retrospective diagnosis than in screening.

The role of the viral load (VL) at birth has been recently investigated to determine whether it could be a reliable marker of disease severity or could predict long-term outcomes; its role in identifying children who will develop complications in their first years of life has also been studied. The VL may be influenced by several factors, such as the timing of intrauterine infection, the PCR assay used, the quality and quantity of immunological response, and potential antiviral treatment [75].

The largest available dataset of blood VL at birth was collected by Marsico et al. in 2018; their study aimed to explore the effect of antiviral therapy on viremia. Among symptomatic newborns, a higher viral load seems to correlate with some markers of active diseases, such as thrombocytopenia and transaminitis [75]. The researchers were unable to find a threshold above which there is a greater risk of SNHL, and they also reported that the individual VL does not predict the hearing outcome; nevertheless, children treated for six months had a higher probability of hearing improvement if their viral load at birth was low. Moreover, the study found a relevant association between higher VL at birth and CNS involvement. This was consistent with a notable study that found statistically relevant differences in quantitative PCR at birth among newborns with moderate-to-severe symptoms and among those with the proven neurological disease [76]. Smilkovic et al. [76] concluded that although there is no VL cut-off to distinguish symptomatic and asymptomatic infants, patients whose baseline viremia was above 100,000 copies/mL had a probability of having moderate-to-severe symptoms that reached 100%. Some other studies suggested that VL might predict late-onset SNHL, although the results came from small population studies with short-term follow-up [77,78].

In summary, the current literature demonstrates a higher baseline VL among children with moderate-to-severe symptoms compared to asymptomatic patients. The role of viral burden in infants with cCMV infection should be further investigated, as it could play a meaningful role in the initial evaluation of newborns with cCMV infection for understanding their risk of permanent sequelae.

## 6. Postnatal Cytomegalovirus Infection in Breastfeeding Neonates

Breast milk (BM) represents the best source of nutrition for newborns, especially if newborns are premature, and exclusive breastfeeding until at least 6 months of postnatal life is recommended by the American Academy of Pediatrics (AAP) [79]. Unfortunately, it is known that BM cannot always be safe, especially due to the possible presence of maternal viruses that can be shed and transmitted to the breastfed newborn. CMV transmission to full-term babies was initially described as a form of natural immunization associated with minimal or no signs of disease [80,81]. Unfortunately, BM-acquired infection can cause severe disease in preterm babies [80]. The elimination of a high viral load, early viral excretion, and a long period of breastfeeding may represent risk factors associated with increased CMV transmission to the child [82,83]. For these reasons, a strategy to remove CMV from BM with a minimal or absent impact on its beneficial components would be desirable. To date, pasteurization, freezing, or ultraviolet-C or microwave irradiation are the available techniques; they show different levels of efficacy and variable effects on BM composition, and many studies are still needed to fully clarify these implications [84].

According to the most recent evidence, fresh BM could be administered only in newborns > 32 weeks of gestational age (GA) or weighing > 1000 g. In the case of more preterm neonates, BM should undergo pasteurization after the first 3 days of lactation in case of maternal positivity for CMV-IgG. However, a recent review suggests that colostrum should also not be considered safe and should not be administered without treatment [84]. In the future, clarification of the real impact of CMV-acquired infection in the neonatal period on the preterm outcome, especially regarding neurological sequelae, would be desirable [85]. Innovative techniques, such as metabolomics, could improve the approach for mother-to-child-transmitted infection [84].

## 7. Prevention

The complex nature of CMV protective immunity, with the possibility of both reactivation of previous infection and the risk of reinfection with genetically distinct viral strains, also produces a major challenge for the development of an effective CMV vaccine.

Ideal vaccines aimed at reducing the impact of congenital CMV infection should have the ability not only to protect seronegative women from primary infection but also to augment the immune response in seropositive women to prevent reactivation or reinfection. Many CMV vaccine candidates are in different stages of investigation with promising results of vaccine candidates found in animal models and transplant recipients. Despite these observations, the prospect of a vaccine for the prevention of maternal and congenital infection does not appear feasible soon [86,87,88,89].

To date, the mainstay of interventions for the prevention of maternal infection and, in turn, of congenital infection, remains the education of pregnant women regarding sources of exposure and behavioral interventions to limit exposure to CMV [16,90].

## 8. Treatment

Advances in neonatal antiviral therapy cCMV infection have been significant in recent years. Indication of treatment is always related to symptomatic disease. Table 2 summarizes the main researches. Studies assessing the treatment of congenital CMV infection began 30 years ago with ganciclovir as the first drug examined, and then oral valganciclovir was used. Because of the toxicity of antiviral drugs, their use in congenitally-infected neonates must balance risks and benefits. Kimberlin et al. of the CASG in 2003 [91] published the results of phase III, randomized, controlled trial of 6-week ganciclovir therapy versus no treatment to determine the effects of this drug on improvement in brainstem-evoked response (BSER) between baseline and 6-month follow-up (or, for patients with normal baseline hearing, normal BSER at both time points). One hundred infants from 1991 to 1999 were enrolled and then randomized to receive six weeks of ganciclovir intravenously at a dose of 6 mg/kg/dose twice daily or to receive no treatment. All study subjects had confirmed isolation of CMV from urine specimens taken within the first month of life. Additionally, the infants enrolled were under 30 days of age, above 32 weeks of gestational age, and had evidence of central nervous system disease, such as microcephaly, intracranial calcification, abnormal cerebrospinal fluid for age, chorioretinitis, and/or hearing deficits. The patient loss rate at follow-up was high, and only 42 infants of 100 had both a baseline and a 6-month BSER audiometric examination. Twenty-one infants (84%) out of twenty-five who received ganciclovir had improved hearing or maintained normal hearing between baseline and 6 months, compared to ten infants (59%) out of seventeen who had taken no treatment. Furthermore, none (0%) of the ganciclovir recipients had hearing deterioration at 6 months versus seven (41%) of seventeen in the control group. Moreover, a total of 43 infants were available for the evaluation of BSER hearing change in the best ear at one year. Significantly fewer patients treated with ganciclovir (21%) had hearing deterioration at one year compared with patients in the control group (68%). In conclusion, although this randomized control trial (RCT) had a high rate of loss to follow-up, it demonstrated that 6 weeks of treatment with intravenous ganciclovir improved hearing outcomes at 6 months and prevented hearing deterioration.

In a subsequent RCT, Oliver et al. [92] tested the effectiveness of ganciclovir therapy in improving neurodevelopmental outcomes at 6 months and 12 months of age compared with patients who did not receive antiviral therapy. The enrolled population was the same as that studied by Kimberlin et al. in 2003 with the same comparative groups: infants who received intravenous ganciclovir for six weeks at a dose of 12 mg/kg/day compared to infants who did not receive treatment. During the course of the previous clinical trial, Denver II developmental tests were performed on subjects at 6 weeks, 6 months, and 12 months of age at the control visits. Denver II is a test used routinely in pediatric care to evaluate developmental milestones and consists of four categories: personal/social, fine motor, language, and gross motor. However, since one of the most important outcomes of congenital CMV infection is hearing loss, the category of language was excluded because the ability to respond to certain stimuli is closely related to the ability to hear. It was demonstrated that ganciclovir therapy might improve neurodevelopmental outcomes at 6 months and 12 months compared to the outcomes of infants who received no antiviral treatment. The largest developmental outcome gap between the infants who received ganciclovir and those who did not receive ganciclovir was in the 12-month assessment. These results, however, did not suggest that treatment with ganciclovir could prevent all neurodevelopmental delays from occurring.

In 2008, a pharmacokinetic/pharmacodynamics study by Kimberlin [93] was published, which showed that 16 mg/kg/dose of valganciclovir, the oral prodrug of ganciclovir, given orally twice daily provided comparable systemic exposure and similar plasma concentration to the administration of intravenous ganciclovir in neonates with symptomatic congenital CMV disease. The toxicity of valganciclovir was similar to that of ganciclovir, with 38% of infants developing moderate or severe neutropenia. The hematologic toxicity and the possible carcinogenicity were justified by the fact that in all these studies, the infants with congenital CMV infection were symptomatic at birth.

Considering that treatment with intravenous ganciclovir for 6 weeks eliminates CMV from the urine, but viruria inevitably returns within 2 weeks of cessation of antiviral therapy, in the 2008–2011 period, the Collaborative Antiviral Study Group (CASG) conducted a phase III, randomized, placebo-controlled trial comparing 6 weeks of oral valganciclovir with 6 months of oral valganciclovir therapy [94]. The study population consisted of 86 symptomatic infants, all 30 days of age or less, with or without central nervous system involvement, such as microcephaly, intracranial calcifications, abnormal cerebrospinal fluid index, chorioretinitis, sensorineural hearing loss, or the detection of CMV DNA in cerebrospinal fluid. Furthermore, eligible participants had a gestational age of 32 weeks or more. In fact, to date, there are no randomized controlled studies that have administered therapy to neonates under 32 weeks of GA with cCMV infection. The results showed that a longer duration of antiviral therapy did not further improve hearing function at the 6-month follow-up compared to the improvements associated with shorter therapy of 6 weeks of oral valganciclovir. However, infants who received 6 months of oral valganciclovir had an improvement in hearing loss at 12 and 24 months (73% compared to 57% of the 6-week group). The timing of initiation of valganciclovir within the first month of life did not correlate with different audiologic outcomes at 12 and 24 months of follow-up. At 24 months, the group receiving 6 months of antiviral therapy also had a better neurodevelopment score estimated with the third edition of the Bayley Scales of Infant and Toddler Development (Bayley-III). Notably, oral valganciclovir was associated with a lower risk of developing neutropenia during treatment compared with the risk associated with intravenous ganciclovir, and in this study, it was shown that the occurrence of neutropenia was similar between the 6-week and 6-month groups (21% versus 27%). Based on the results of this RCT, antiviral treatment for 6 months is considered an effective and well-tolerated option for symptomatic infants to improve hearing and neurodevelopment long-term outcomes. The cited study [94] also explored how the viral load decreased in blood during antiviral treatment and what differences exist between the two groups. In the analysis, lower viral loads compared with higher viral loads correlated with better hearing function at 6, 12, and 24 months of follow-up among infants in the 6-month antiviral therapy group. In contrast, at 6 weeks, oral valganciclovir did not show a correlation between viral load and a better endpoint.

Subsequently, in 2016 in a retrospective study, Bilavsky et al. [95] observed that in infants with symptomatic cCMV infection who started antiviral treatment during the first 4 weeks of life, receiving this treatment for 12 months significantly improved hearing status. This study revealed that in most ears with mild or moderate hearing loss at birth, hearing improved, returning to normal (64.9% of the affected ears improved, and 6.5% deteriorated; 76% of the improved ears returned to normal hearing). Even in cases of severe hearing loss at birth, 40% of ears benefited from antiviral treatment [95]. Furthermore, the probability of hearing improvement seemed to be inversely related to the severity of impairment at birth. This study raised a simple but interesting question as to how antiviral treatment generates improvement rather than preventing the deterioration of SNHL. In the literature, an answer has not yet been found. However, it is speculated that the dynamic process of infection and inflammation due to the virus continues to occur even after birth [95].

## 9. Side Effects

It is important to address the potential toxicity associated with antiviral drugs. The main side effects include significant neutropenia; this is less common with oral valganciclovir than with intravenous ganciclovir and generally occurs in the first month of treatment [94]. No increased toxicity was observed in RCTs evaluating 6 months versus 6 weeks of treatment. In addition, the administration of oral valganciclovir eliminates central line complications and hospitalization-related risks. Other side effects observed are thrombocytopenia and hepatotoxicity, reported in up to 30% of patients treated with ganciclovir. The possible appearance of these side effects may require the interruption of therapy [96]. Considering short-term toxicity, it is important to monitor patients regularly with clinical and blood examinations.

Long-term side effects and safety of administration of antiviral therapy for cCMV infection to infants are still not well established. However, gonadal toxicity and carcinogenicity have been observed in animal models. Further studies are needed to establish possible long-term complications of this treatment strategy. Antiviral drugs with better toxicity profiles are required to justify the risk versus benefit of treatment in asymptomatic infected babies who suffer CMV-associated hearing loss [97].

## 10. Conclusions

In the pediatric population, cCMV infection is the first non-genetic cause of SNHL, as well as a relevant cause of other morbidities in neonates. Despite the promising developments in neonatal diagnosis and antiviral therapies for cCMV over the last few years, there are still some unanswered questions. Preventive interventions other than behavioral measures during pregnancy are still lacking, although there is a large amount of research interest on this subject, and many clinical trials are currently ongoing to formulate a vaccination for pregnant women. Currently, we recommend using a PCR assay in blood, urine, and saliva rather than culture for the diagnosis of cCMV infection since PCR does not require expensive tissue culture; it is a more rapid test and is associated with a relatively low cost. A universal screening test using DBS, a routine specimen collected at birth, has been proposed but still requires further investigation and development for widespread use. We restate the importance of appropriate storage of the specimen taken at birth to permit us to distinguish a congenital infection from a postnatal infection, attributable, for instance, to infected BM from a seropositive mother [64,98].

At present, there is no evidence of the benefit of antiviral therapy in asymptomatic infants. The use of antiviral therapy must take into account known risks, such as neutropenia and/or thrombocytopenia, and possible risks, such as gonadal toxicity and carcinogenicity, which have been observed in animal models, in addition to all potential benefits [97]. We actually recommend treatment for cCMV infection with oral valganciclovir (16 mg/kg/dose twice a day) for a duration of 12 months. The effectiveness and tolerability of this therapy option have proven effective for hearing and neurodevelopmental long-term outcomes. Valganciclovir is reserved for congenitally-infected neonates with the symptomatic disease at birth, such as microcephaly, intracranial calcifications, abnormal cerebrospinal fluid index, chorioretinitis, or sensorineural hearing loss. Treatment with antiviral drugs is not routinely recommended for neonates with the mildly symptomatic disease at birth, for neonates under 32 weeks of gestational age, or for infants more than 30 days old because of insufficient evidence from studies. However, since these populations represent the vast majority of neonates and infants with cCMV infection and they are at risk of developing late-onset sequelae, a biomarker able to predict long-term sequelae should also be found to justify starting treatment and reducing the burden of CMV-related complications.

## Figures and Tables

**Table 1:** Possible signs and symptoms in children with congenital cytomegalovirus (cCMV).

**Clinically Detectable Symptoms/Signs**
**Physical examination**
Small for gestational age (birth weight ≤ 2 standard deviations for gestational age)
Microcephaly (head circumference ≤ 2 standard deviations for gestational age)
Petechiae or purpura (usually found within hours of birth and persist for several weeks)
Blueberry muffin rash (intradermal hematopoiesis)
Jaundice *
Hepatomegaly
Splenomegaly
**Neurologic physical examination**
Microcephaly (head circumference ≤ 2 standard deviations for gestational age)
Neurologic signs (lethargy, hypotonia, seizures, poor sucking reflex)
**Abnormalities Detected Incidentally or Through Subsequent Investigation/Specialist Examination**
**Laboratory results**
Anemia
Thrombocytopenia (occurs in the first week, but platelets often increase spontaneously after the second week)
Leukopenia, isolated neutropenia
Elevated liver enzymes (alanine aminotransferase/aspartate aminotransferase)
Conjugated hyperbilirubinemia
**Cerebrospinal fluid**
Abnormal cerebral fluid indices, positive CMV DNA
**Neuroimaging**
Calcifications, periventricular cysts, ventricular dilatation, subependymal, pseudocysts, germinolytic cysts, white matter abnormalities, cortical atrophy, migration disorders, cerebellar hypoplasia, lenticulostriatal vasculopathy
**Hearing test**
Sensorineural hearing loss uni- or bilaterally
**Visual examination**
Chorioretinitis, retinal hemorrhage, optic atrophy, strabismus, cataracts

* CMV-associated jaundice can be present on the first day after birth and usually persists longer than physiologic jaundice.

**Table 2:** Main studies on therapy of symptomatic congenital cytomegalovirus (cCMV) infection.

Study	Details	Results	Findings
Kimberlin et al. [91]	44 patients evaluated with confirmed isolation of CMV from a urine specimen obtained before study enrolment and within the first month of life, and all had evidence of CNS disease, such as (1) microcephaly; (2) intracranial calcifications; (3) abnormal cerebrospinal fluid (CSF) for age; (4) chorioretinitis; and/or (5) hearing deficits. Infants ≤ 1 month of age, ≥32 weeks’ gestation, and weighing ≥ 1200 g at birth were eligible for study participation. 6 weeks therapy with IV ganciclovir vs. no treatment.	Improved or maintained normal hearing between Baseline and 6 months: 84% GCV vs. 58% controls (*p* = 0.06). Baseline and 12 months: 79% GCV vs. 32% controls (*p* = 0.133). Worsening of hearing between Baseline and 6 months: 0% GCV vs. 41% controls (*p* < 0.01). Baseline and 12 months: 21% GCV vs. 68% controls (*p* < 0.01).	IV ganciclovir for 6 weeks prevented hearing deterioration at 12 months in patients with CNS disease.
Kimberlin et al. [93]	24 patients with symptomatic cCMV disease: culture confirmation of CMV in urine or throat swab specimens within 30 days of birth; symptomatic congenital CMV disease or CNS involvement; the age of <30 days; body weight ≥ 1200 g. and gestational age ≥ 32 weeks. antiviral therapy for 6 weeks with VGC oral solution vs. intravenous GCV.	16 mg/kg/dose of VGC given orally twice daily reliably provided comparable systemic exposure to GCV.	Oral VGC had comparable efficacy to intravenous GCV with fewer short-term side effects.
Oliver et al. [92]	100 neonates with cCMV involving the CNS. 6 weeks of intravenous ganciclovir vs. no treatment. Denver development test at the ages of 6 weeks, 6 months, and 12 months: numbers of milestones did not meet, “delays” were determined.	Numbers of delays 6 weeks: 1.5 GCV vs. 2.05 controls (*p* = 0.11). 6 months: 4.46 GCV vs. 7.51 controls (*p* = 0.02). 12 months: 10.06 GCV vs. 17.14 controls (*p* = 0.007).	Improved developmental delay at both 6 and 12 months in infants who had received treatment compared to infants who did not receive treatment.
Kimberlin et al. [94]	87 neonates with symptomatic congenital CMV disease (with or without CNS involvement), age ≤ 30 days; gestational age ≥ 32 weeks, and body weight ≥ 1800 g at the initiation of therapy. 6 months vs. 6 weeks therapy with valganciclovir (16 mg/kg orally twice daily)	No significant findings in between-group differences in change in best-ear hearing and in total-ear hearing between baseline and 6 months. Change in total-ear hearing between baseline and 12 months and 24 months differed significantly between the two groups. Communicative endpoints of scores on the language-composite component and the receptive communication scale of the Bayley-III assessment were improved with the longer treatment. Incidence of grade 3 or 4 neutropenia was similar among participants assigned to continue valganciclovir and those randomly assigned to receive a placebo.	6 months of treatment with VGC did not improve hearing at 6 months but did improve hearing and neurodevelopment in the long term (at 12–24 months).
Bilavsky et al. [93]	Infants with symptomatic cCMV, who started antiviral treatment during the first 4 weeks of life and had at least 1 year of follow-up.	Hearing impairment at birth was found in 54 (36.2%) of the 149 infants diagnosed with symptomatic cCMV (77 affected ears). After 1 year of antiviral treatment and a long-term follow-up of the 77 affected ears at baseline, 50 (64.9%) improved, 22 (28.6%) remained unchanged, and 5 (6.5%) deteriorated. Most improved ears (38/50 = 76%) returned to normal hearing. Improvement was most likely to occur in infants born with mild or moderate hearing loss and less likely to occur in those with severe impairment.	Infants born with cCMV and hearing impairment, receiving 12 months of antiviral treatment, showed significant improvement in hearing status. The probability of hearing improvement seemed inversely related to the severity of the impairment at birth.

CNS, central nervous system; GCV, ganciclovir; VGC, valganciclovir.
